# Institutional review boards: consideration in developing countries.

**DOI:** 10.3201/eid0707.017726

**Published:** 2001

**Authors:** J W Pape

**Affiliations:** Cornell University Medical College, New York, New York 10021, USA. jwpape@gheskio.org


emphasis on partnerships, decision-making by African scientists, and a strong scientific basis for the funded research. To support a variety of research programs, MIM has also developed the Malaria Research and Reference Reagent Resource Center, which provides high quality reagents and materials to investigators who are, or wish to be, involved in malaria research. NIH's National Library of Medicine has taken responsibility for enhancing the capacity of African scientists to do research by establishing and supporting access to communications and information resources. A number of research networks are online using very small aperture telecommunications (VSAT) technology for Internet access. This allows for shared databases, electronic mail and discussion groups, access to published literature, and use of remote sensing technologies. Information about the progress of MIM is shared through meetings, a newsletter, and on the internet at http://mim.nih.gov

Future goals of MIM include stabilizing funding for the MIM-TDR grant program, developing new partnerships, and creating new training opportunities, such as training on research management. Scientific research on *Plasmodium vivax* and on malaria-related anemia is being conducted. Interactions with RBM are well-established and coordinated. The 2nd International MIM Conference is scheduled for 2002 in Tanzania.

---

### Institutional Review Boards: Consideration in Developing Countries

**Jean William Pape**
Cornell University Medical College, New York, New York, USA, and Groupe Haitien d'Etude du Sarcome de Kaposi et des Infections Opportunistes, Port-au-Prince, Haiti

Institutional review boards (IRBs) play an essential role in protecting the rights of volunteers involved in research projects. Their function has become more complex, particularly concerning projects conducted in developing countries. But can IRBs in the United States guarantee the protection of human subjects involved in research projects in developing countries?

IRBs have no effective way of controlling what goes on in the field. The complex ethical clearance process does not determine whether persons engaged in research projects in developing countries are fully aware of the major aspects of the studies they participate in. The clearance process includes the IRB approval and consent forms. Required U.S. consent forms are too long and the language too complicated to be certain all participants have a full understanding of the study. The forms also appear to be intended more to offer legal protection to sponsoring agencies than to protect the welfare of the volunteer. Most importantly, the forms do not guarantee that volunteers have fully understood the objectives, risks, and benefits of the study and the extent of their voluntary participation. To protect volunteers as well as all persons and institutions involved, these forms must not only communicate necessary information concerning the study to be conducted but also evaluate volunteers' knowledge and their desire to participate. To achieve this goal, we propose to use a simple questionnaire administered by a team not involved in the volunteer recruitment process. We have used such a questionnaire to evaluate potential volunteers for a phase-II HIV vaccine trial. Although volunteers had three intensive, 2-hour counseling sessions, only half responded correctly to all 21 questions. The others were referred for additional counseling and reevaluation.

The IRB process requires that collaborative projects with U.S. institutions have clearance from multiple IRBs. Each IRB meets generally once a month and uses its own consent forms. Each has its own set of rules. Each will respond with different concerns that must be addressed. The approval process may create a lag time of 3 to 12 months to obtain ethical clearances for a project lasting 12 to 24 months.

The ethical clearance process can be simplified in several ways: 1) All studies supported by NIH should have a unique IRB application form and a unique IRB consent form. 2) A certain percentage of the research grant should be allocated to support the ethical clearance process. Ethical support should be available at the grant's initiation. 3) While waiting for the formal ethical clearance and final consent, potential volunteers could be counseled and evaluated. 4) The primary responsibility of local and national IRBs should be clearly determined. IRBs must share responsibilities to achieve the greatest benefit for volunteers. 5) A mechanism must be developed to resolve conflicts between IRBs from developed and developing countries. Yearly meetings of IRBs from host and sponsoring institutions should take place to facilitate the exchange of documents and other information.

Address for correspondence: Jean William Pape, Division of International Medicine and Infectious Diseases, Cornell University Medical College, Room A-421, 1300 York Avenue, New York, NY 10021 USA; fax: ; e-mail: jwpape@gheskio.org

